# Tonsuring: Myths and facts

**DOI:** 10.4103/0974-7753.51927

**Published:** 2009

**Authors:** Kaliaperumal Karthikeyan

**Affiliations:** Assistant Professor, Department of Dermatology & Venereology, Sri ManakulaVinayagar Medical College, Pondicherry

**Keywords:** Hair, religion, tonsuring

Tonsuring is the act or process of cutting the hair, especially as a religious rite or custom.[1] Tonsuring is a fashionable practice in many races. But the origin of the tonsure is something of a mystery. Early Celts, a people based in northern Britain, were thought to have worn the tonsure prior to their contact with the Roman Empire and with no relation to religion. Members of both the Eastern Orthodox Church and the Roman Catholic Church wore the tonsure, and both claim that its origins go back to the time of Jesus Christ. In the Latin or Western Rite of the Roman Catholic Church, 'first tonsure' (generally consisting of a symbolic cutting of a few tufts of hair or at most a coin-sized bare spot toward the back of the head) was the rite of inducting someone into the clergy. There are different types of tonsures based on the pattern such as clerical, baptismal, and monastic. The practice was in vogue till the Roman Catholic Church abolished the practice of tonsure in 1972.

Tonsuring is also a religious ceremony in Hindu religion. According to the rules of the Vedas, the Chudakarana (tonsuring of hair) should be performed either in the first or the third year of the child. It is practiced even today in most Hindu communities. In Buddhism, tonsure is a part of the rite of becoming a monk. This involves shaving the head and face. This tonsure is renewed as often as required to keep the head cleanly shaven, and some Chinese Buddhist monks also have 6, 9, or 12 dots on the top of the head as a result of burning the shaven scalp with the tip of a smoking incense stick. In Islam, it is often customary for pilgrims on the Hajj to shave their heads before entering Mecca as a sign of their rejection of vanity and for cleanliness. Jain monks pluck their hair so as to keep their scalp bare and devoid of lice. They do not use blade or knife.

Tonsuring is a common ritual rite in the temple town of Tirupathi (South India) and daily both men and women tonsure in thousands. Hindu devotees offer their hair to Lord Balaji for favors received, to show gratitude and respect. Both men and women offer their hair. It is interesting to note that more than 1,500 women partake in the ceremony daily. Temple authorities sell the hair thus obtained. In 2007, Tirumala Tirupati Devasthanams sold human hair worth 450 million Indian rupees.[2] The long hair of women is more priced and used in making hair extensions and wigs. The tonsured hair from men is used to extract l-cysteine. It is a precursor in the food, pharmaceutical, and personal care industries. One of the largest applications is the production of flavors.[3]

The common myth prevalent among public is that shaving of hair increases the hair growth. The effect of repeated shaving on human hair growth was studied by Lynfield and Macwilliams.[4] In their study, five healthy young white men each shaved one leg weekly for several months and left the other leg as a control. No significant differences in total weight of hair produced in a measured area, or in width or rate of growth of individual hairs, could be ascribed to shaving. The probable reasons for the misconception could be the shortness of the shaft of a shaved hair allows changes in its length to be noticed more easily. Taking the example of the male with a full beard, it is impossible to detect the daily increase in beard growth. But it is a different story with the clean-shaven person whose bristle growth can be seen on the very same day. The short, shaved hairs being held more erect by the follicles clasped firmly around them cause apparent coarseness. Tonsuring can also be therapeutic in curing many diseases such as pediculosis, plica polonica, and peidra. Tonsuring can also be associated with secondary bacterial infections if clean blades are not used. Further tonsuring is associated with the risk of transmitting HIV and Hepatitis B virus infection if the blades are reused without sterilizing.
